# HSRA: Hadoop-based spliced read aligner for RNA sequencing data

**DOI:** 10.1371/journal.pone.0201483

**Published:** 2018-07-31

**Authors:** Roberto R. Expósito, Jorge González-Domínguez, Juan Touriño

**Affiliations:** Computer Architecture Group, Universidade da Coruña, Campus de Elviña, 15071 A Coruña, Spain; University of Helsinki, FINLAND

## Abstract

Nowadays, the analysis of transcriptome sequencing (RNA-seq) data has become the standard method for quantifying the levels of gene expression. In RNA-seq experiments, the mapping of short reads to a reference genome or transcriptome is considered a crucial step that remains as one of the most time-consuming. With the steady development of Next Generation Sequencing (NGS) technologies, unprecedented amounts of genomic data introduce significant challenges in terms of storage, processing and downstream analysis. As cost and throughput continue to improve, there is a growing need for new software solutions that minimize the impact of increasing data volume on RNA read alignment. In this work we introduce *HSRA*, a Big Data tool that takes advantage of the MapReduce programming model to extend the multithreading capabilities of a state-of-the-art spliced read aligner for RNA-seq data (HISAT2) to distributed memory systems such as multi-core clusters or cloud platforms. *HSRA* has been built upon the Hadoop MapReduce framework and supports both single- and paired-end reads from FASTQ/FASTA datasets, providing output alignments in SAM format. The design of *HSRA* has been carefully optimized to avoid the main limitations and major causes of inefficiency found in previous Big Data mapping tools, which cannot fully exploit the raw performance of the underlying aligner. On a 16-node multi-core cluster, *HSRA* is on average 2.3 times faster than previous Hadoop-based tools. Source code in Java as well as a user’s guide are publicly available for download at http://hsra.dec.udc.es.

## 1 Introduction

RNA sequencing (RNA-seq) [1, 2] stems from the application of Next Generation Sequencing (NGS) technologies to complementary DNA molecules, which are obtained by reverse transcription from a single stranded RNA (e.g., messenger RNA). RNA-seq analysis is mainly used to get information about the presence and quantity of RNA in a biological sample at a given moment. Nowadays, RNA-seq is becoming an increasingly efficient and popular tool for quantifying gene expression levels and identifying variants in the transcriptome, providing much higher resolution measurements of gene expression than other methods such as hybridization-based microarrays [3].

The rapid advance of high-throughput NGS technologies has led to a vast production of short DNA sequence fragments (called reads) with dramatically low unit cost. A typical RNA-seq data analysis begins by mapping these reads to a given reference genome to determine the location from which the reads were originated. This early mapping step is considered a fundamental part to nearly all NGS workflows, and the accuracy of downstream analyses depends heavily on it. However, optimally aligning hundreds of millions of reads to multiple gigabases for the typical human genome (the most common use case) is one of the most computationally intensive steps in the entire process. Therefore, the explosive growth of RNA-seq datasets poses a big challenge to the mapping quality and the execution speed of existing spliced aligners. Even though state-of-the art tools can provide high accuracy and speed, the mapping step will remain very time-consuming as NGS technologies and their associated costs are expected to continue to improve over time, which can represent a significant bottleneck in future RNA-seq analyses.

Such growth in the amount of genomic data can be tackled by taking full advantage of high-performance approaches based on parallel and distributed data processing techniques that scale efficiently with the number of computing nodes. Although most of the existing spliced aligners for RNA-seq data include native support for multithreading to exploit the computational capabilities of current multi-core systems, their scalability is inherently limited to a single computing node. To overcome this issue, popular Big Data technologies like the MapReduce paradigm [4] provide efficient support for the distributed storage and processing of massive datasets. Such Big Data frameworks are capable of composing large distributed applications which can be executed on commodity clusters and cloud platforms in a scalable way. In fact, the use of Big Data and MapReduce are gaining increasing attention in bioinformatics and biomedical research in recent years [5–8].

In this paper we introduce *HSRA*, a spliced read aligner that relies on the MapReduce model to enable scalable mapping of very large RNA-seq datasets on distributed memory systems. Our tool is intended for those bioinformatics researchers who perform their RNA-seq analyses using Big Data platforms and frameworks [9]. *HSRA* allows them to efficiently distribute their mapping tasks over the nodes of a computing cluster or cloud platform by combining a fast and accurate multithreaded spliced aligner (HISAT2 [10]) with the Apache Hadoop project [11], which is the most popular open-source MapReduce framework for distributed data processing. *HSRA* currently supports single- and paired-end read alignments in FASTQ/FASTA formats and is capable of directly processing input datasets compressed with gzip and bzip2 codecs.

The remainder of the paper is organized as follows: Section 2 introduces the background of the paper. Section 3 discusses the related work. The design and implementation of our tool is described in Section 4. Section 5 presents the experimental results carried out on a multi-core cluster to evaluate the performance of our proposal. Finally, Section 6 concludes the paper and proposes future work.

## 2 Background

This section describes the main concepts and involved technologies that *HSRA* relies on: short read alignment (Section 2.1) and MapReduce (Section 2.2), which are necessary to understand the design and implementation of the tool.

### 2.1 Short read alignment of RNA-seq data

After quality control (e.g., filtering out low quality reads), the fundamental task in RNA-seq analyses is mapping each read to a previously assembled reference genome or transcriptome. In the context of RNA-seq, mapping to a reference genome is the preferred choice as it is much more effective for the identification of novel genes or transcripts [12]. So far, many algorithms have been proposed in the literature to perform short read alignment to a reference genome, such as BWA [13], Bowtie [14], Bowtie2 [15], MAQ [16], RMAP [17] and SOAP2 [18]. Nevertheless, the complexities inherent to RNA-seq data make RNA-seq alignment much more challenging than mapping DNA-seq data. As genes in eukaryotic organisms contain introns and because RNA-seq reads do not include these introns, many reads may span two or more exons. Conventional mapping algorithms are not recommended because of their inability to align reads to the genome across splice junctions. One approach to resolve this issue is to supplement the reference genome with reads derived from exon-exon splice junctions acquired from known gene annotations [19]. A preferred strategy is to use specialized splice-aware aligners to perform this critical step that can recognize the difference between a read aligning across an exon-intron boundary and a read with a short insertion [12]. Other important ability of RNA-seq aligners must be to perform gapped alignment to handle reads containing sequencing errors or indels [20]. Finally, it is also worth mentioning lightweight-alignment or pseudoalignment-based approaches used by some recent RNA-seq aligners such as Salmon [21] and kallisto [22].

#### 2.1.1 State-of-the-art splice-aware aligners

A big challenge in RNA-seq analyses is to choose a right mapping tool among existing ones that is capable of: (1) aligning reads across splice junctions; (2) performing gapped alignment; (3) handling paired-end reads for higher accuracy; and (4) running efficiently both in terms of execution time and memory consumption.

A recent comprehensive study has evaluated 14 common splice-aware aligners for RNA-seq data [23], most of them meeting the first three requirements. Considering only open-source tools, state-of-the-art aligners that were evaluated include GSNAP [24], STAR [25], SOAPSplice [26], MapSplice [27], TopHat2 [28] and HISAT2 [10]. According to [23], TopHat2 has been the most popular aligner over the last 5 years, mainly due to high sensitivity and accuracy of mapping. However, it is among the slowest aligners in terms of mapping speed while showing moderate memory consumption. TopHat2 is now being largely superseded by HISAT2, which is expected to inherit its popularity in the near future as it provides the same core functionality in a more accurate and much more efficient way. The default mode of HISAT2 follows a novel hybrid approach that collects splice sites as it processes the reads, similarly to the first run of two-pass methods (e.g., TopHat2, STAR). Those splice sites are used when aligning later reads in the same run, which allows to increase sensitivity without the large performance cost incurred by two-pass methods. By using hierarchical indexing (i.e., global and local indexes) and several alignment strategies, HISAT2 is currently the fastest tool while remarkably accurate even on the shortest anchors and without annotation [23]. In terms of memory consumption, HISAT2 has very low memory requirements (4.3 GiB for the human genome [10]) as it is based on an extension of the Burrows-Wheeler Transform (BWT) for graphs [29] instead of using more memory-consuming hash-based or suffix array approaches. Note that according to this study, other tools are either considerably slower than HISAT2 (SOAPSplice), slighlty slower but much more memory-consuming (STAR, GSNAP), or both (MapSplice). Therefore, we have selected HISAT2 as the underlying aligner for *HSRA* in order to implement the fastest and lowest memory-consuming distributed tool. HISAT2, as well as most of the mapping tools, provides its own parallel implementation through multithreading to support shared memory systems, so its scalability is limited to a single node.

### 2.2 MapReduce

**Fig 1:**
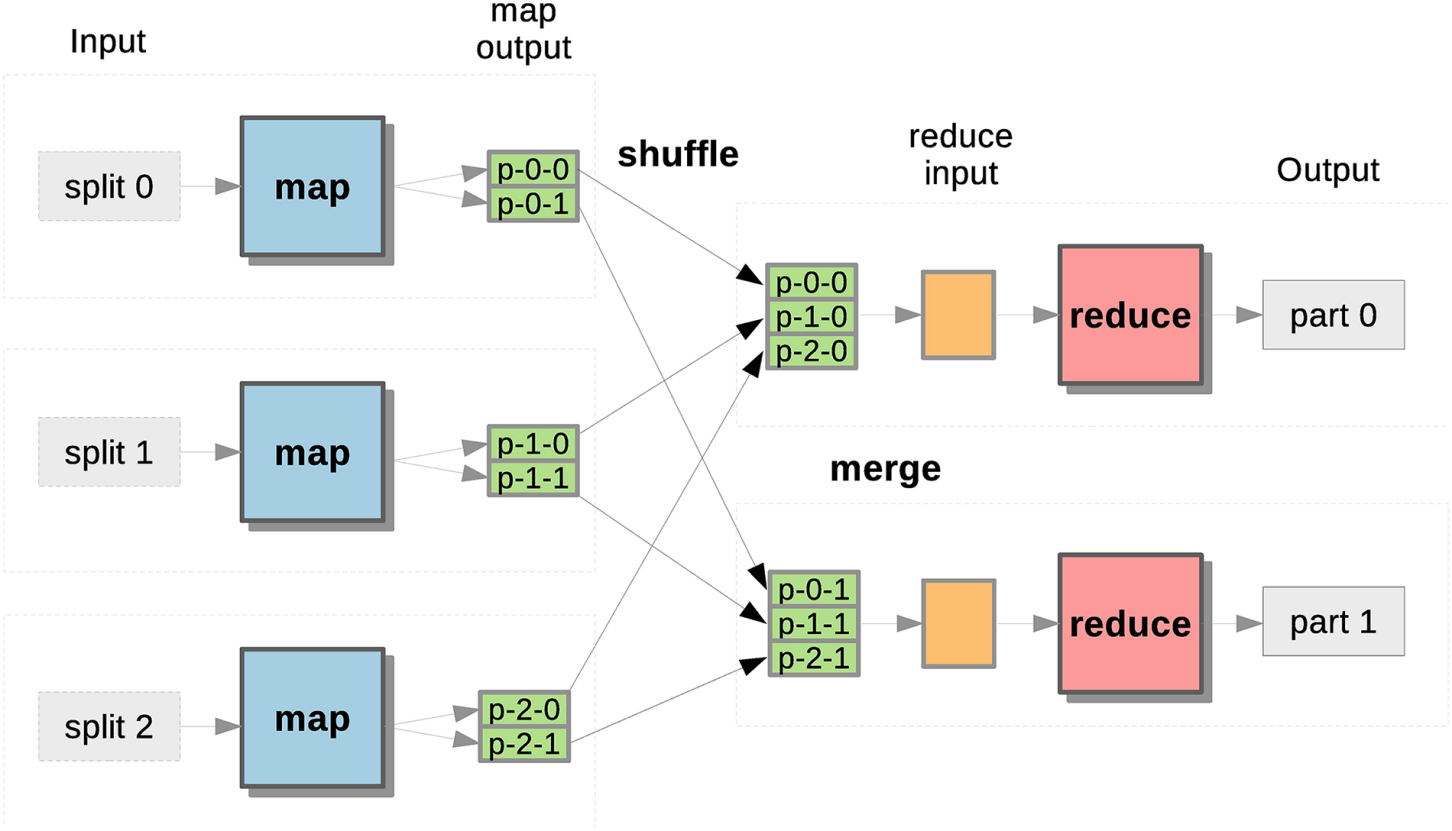
Overall workflow of the MapReduce paradigm. This workflow shows several map and reduce tasks working in parallel over different input splits.

MapReduce is a parallel programming model originally developed by Google [4] for the storage and processing of large datasets over the nodes of distributed memory systems such as clusters and clouds. In fact, it is one of the most successful paradigms for effective Big Data processing in many industrial and scientific fields. Other popular parallel programming models such as the Message Passing Interface (MPI) [30] require developers to explicitly manage inter-process communications. Instead, MapReduce allows transparent parallelization by means of two explicit user-defined functions derived from functional programming: *Map* and *Reduce*. The basic idea of this model is shown in Fig 1. The input dataset to be processed is divided into splits or chunks, each one containing many records in a <key, value> pair format. The *Map* function transforms the input key-value pairs into other intermediate ones based on any relationship specific to the application. When the input is large, many instances of the *Map* function (i.e., map tasks) can execute in parallel on different input splits (i.e., one map task per split). Once the map tasks are completed, the intermediate key-value pairs are sorted and grouped together according to the key by the MapReduce framework. Then, the framework shuffles all these data across the network so that all the values with the same key are merged together into a single list, which is the input of the *Reduce* function. Several instances of the *Reduce* function (reduce tasks) can be executed concurrently, whose number is configurable by the user. The reduce tasks produce the final output in the form of key-value pairs. Unlike MPI, all the inter-process communications between mappers and reducers (i.e., data shuffling) are completely transparent, as well as other mechanisms such as resource management and fault tolerance. In this model, users only need to focus on implementing the *Map* and *Reduce* functions.

MapReduce has been specifically designed for the scalable processing of very large datasets, far beyond what can be stored in memory. In order to efficiently support this model, Google developed the distributed Google File System (GFS) [31], designed to provide high bandwidth by replicating and partitioning files across the locally attached disks of the computing nodes. Basically, the number of times that GFS replicates each data block over the cluster is defined as the replication factor. Relying on GFS, MapReduce attempts to schedule map tasks on the nodes where the input data blocks reside, improving data locality and minimizing data movements across the network. It is important to remark that the MapReduce model can support map-only applications, where no reduce tasks are executed (i.e., no grouping-by-key operation is performed). For these applications, the output of map tasks is the final output. Avoiding the reduce phase eliminates sort and shuffle phases as well, which reduces disk and network overheads. The intermediate output of map tasks is generally written to local disk before being sent to the reducers, but in map-only applications this output is directly written to GFS.

#### 2.2.1 Apache Hadoop

The Apache Hadoop project [11] is the most popular and widespread open-source implementation of the MapReduce model derived from the Google’s proprietary one. Basically, Hadoop consists of three components or layers: (1) the Hadoop MapReduce engine as data processing layer; (2) the Hadoop Distributed File System (HDFS) [32] as storage layer that mimics GFS; and (3) the Yet Another Resource Negotiator (YARN) [33] as resource management layer. Hadoop is entirely written in Java to ensure high portability and is widely used in both academy and industry. In addition to on-premises deployments, Hadoop is becoming a *de facto* standard for cloud computing platforms, where storage and compute resources can be accessed on demand on a pay-as-you-go basis.

In order to extend the multithreading capabilities of the spliced HISAT2 aligner to distributed memory systems, *HSRA* has been implemented on top of Hadoop due to its interesting features such as scalability, portability, distributed data management, fault tolerance and data-aware scheduling, as well as its high popularity and support in the Big Data ecosystem.

## 3 Related work

There are in the literature some previous works that exploit parallel architectures to speed up the performance of the alignment procedure. This section provides a state-of-the-art survey focused on those mapping tools for DNA (Section 3.1) and RNA (Section 3.2) sequencing data intended to be executed on distributed memory systems. The goal is to gather the major limitations and main causes of inefficiency of previous tools in order to avoid them, to the extent possible, when designing *HSRA*.

### 3.1 Distributed mapping tools for DNA-seq data

Most of the distributed tools for DNA are based on non-spliced aligners (e.g., BWA, Bowtie, RMAP), which are not recommended for mapping RNA-seq data as mentioned in Section 2.1. Representative examples of such tools are pBWA [34], parSRA [35], CloudBurst [36], SEAL [37], CloudAligner [38], BigBWA [39], SparkBWA [40], Halvade [41] and Crossbow [42], briefly described next.

pBWA is implemented following the MPI paradigm, but it is limited to a particular and outdated version of the BWA aligner, while its scalability has proved to be rather poor [35]. parSRA is a novel framework that can work with several underlying mapping tools (e.g., Bowtie2, BWA, SOAP2). However, the current version does not provide support for any splice-aware aligner. Furthermore, parSRA is based on the UPC++ [43] parallel extension of C++ and the FUSE kernel module, which are requirements not frequently available on clusters (the FUSE module cannot be installed by regular users). The rest of tools are based on Big Data technologies, mainly Hadoop, but they generally present some limitations and shortcomings. CloudBurst implements the RMAP algorithm but it does not support paired-end reads and the commonly used FASTQ format for input sequence files. SEAL only works with a particular and modified version of BWA. Regarding CloudAligner, it is an RMAP-based tool that requires a preprocessing of the genome index and the input sequence files before being copied to HDFS, incurring high overhead. Note that some kind of preprocessing or conversion of the sequence files is generally required for all other Hadoop-based tools (i.e., CloudBurst, SEAL), which prevents any of them from processing FASTQ/FASTA datasets (compressed or not) directly from HDFS. This is also the case for the Hadoop-based BigBWA tool. Even worse, the output files of the underlying BWA aligners executed by BigBWA are first stored in local disk and then copied to HDFS, incurring high disk overhead. SparkBWA outperforms BigBWA by using Apache Spark [44], but it cannot process compressed datasets either and does not support the FASTA format for input sequence files. Halvade follows a different approach by implementing a whole genome analysis pipeline instead of only the alignment step. Implemented with Hadoop, it performs read alignment supporting several tools (BWA, Bowtie2) and variant calling using the appropriate modules from the Genome Analysis Toolkit (GATK) [45]. However, the performance of the alignment step in Halvade is similar to previous tools such as BigBWA according to [40]. The major issues that hinder its performance are: (1) Halvade also requires a preprocessing of the paired-end input files to adapt them to the format required by Hadoop-BAM [46], by interleaving both files so that paired-end reads are adjacent to each other; (2) a preparatory step (partitioning) of the reference genome is also required; and (3) for some aligners (e.g., Bowtie2), Halvade incurs significant disk overhead as it first copies the input reads parsed from HDFS to local disk from which the underlying aligner will then perform the mapping procedure. Finally, Crossbow follows a similar approach to that of Halvade, also implementing a whole analysis pipeline using Hadoop but providing significantly lower parallel efficiency according to [41].

### 3.2 Distributed mapping tools for RNA-seq data

There also exist a few distributed tools specifically intended for mapping RNA-seq data. The most important projects are pMap [47], FX [48], Myrna [49], DistMap [50] and Halvade-RNA [51], briefly described next.

pMap is an MPI-based tool that can use several underlying aligners. Among them, GSNAP is the only one that can be used for RNA-seq data, although it is slower and much more memory-consuming than HISAT2 as mentioned in Section 2.1.1. Moreover, pMap suffers from one major issue that severely limits its scalability: the overhead of the initial partitioning and distribution of the input sequence files is significant, especially when increasing the number of nodes, as stated in [35]. Another GSNAP-based tool is FX, implemented with Hadoop, but currently unavailable to researchers (the website of this project is not longer accessible). Furthermore, FX also requires a preprocessing step before aligning paired-end reads, which converts the FASTQ input files to the custom GSNAP format [24]. Myrna is another Hadoop-based tool that calculates the differences of gene expression in RNA-seq datasets. So, this tool is not just an aligner, but instead integrates several functions for RNA-seq analysis such as normalization and statistical modeling in a single computing pipeline. Myrna uses Bowtie [14] as the underlying aligner, which is neither splice-aware nor performs gapped alignment. Although Myrna is intended for RNA-seq data, its main limitation is that expression signal may be lost as the alignment step cannot align reads across exon junctions [5, 49]. Regarding DistMap, it provides an integrated workflow implemented in a series of Perl scripts that run on Hadoop using the streaming interface. It supports a wide range of aligners and, among them, GSNAP, STAR and TopHat2 are suitable for RNA-seq data, thus lacking support for the faster and more memory-efficient HISAT2. DistMap also incurs significant preprocessing overhead as it converts the input FASTQ files into appropriate file formats capable of being processed with the default record readers provided by Hadoop. Moreover, it suffers from significant disk overhead during the mapping procedure as it also copies the input reads from HDFS to local disk in a similar way to Halvade. In addition to this, DistMap does not support the FASTA format for input sequence files. Finally, Halvade-RNA is an extension of Halvade that provides a whole analysis pipeline for RNA-seq data using STAR as the underlying aligner. Therefore, the same limitations arise as for Halvade. In fact, a significant disk overhead is incurred when using STAR as in the case of using Bowtie2 in Halvade.

The main goal of *HSRA* is to provide scalable read alignment for RNA-seq data analyses. To do so, *HSRA* integrates the fast spliced aligner HISAT2 into Hadoop. Furthermore, *HSRA* tries to avoid the main limitations found in previous tools. To the best of our knowledge, *HSRA* is the first publicly available distributed tool that performs short read alignment based on HISAT2.

## 4 Materials and methods

Section 3 has revealed the most common issues and causes of inefficiency in previous distributed mapping tools. These limitations can be converted into desirable requirements when implementing *HSRA*, which can be summarized as follows:

1. The tool must be based on a fast multithreaded, but accurate enough, splice-aware aligner with low memory requirements.
2. The tool must support both single- and paired-end read alignment of input datasets in common unaligned sequence formats (e.g., FASTQ, FASTA).
3. The tool must provide the output alignments in *de facto* standard formats (e.g., SAM/BAM [52]) without any additional conversion to enable direct interoperation with downstream analytical tools (e.g., GATK [45]).
4. The tool must work with an unmodified version of the selected aligner.
5. The tool must not be limited to working with a particular version of the aligner, and should support any future version (to the extent possible).
6. The tool must support the processing of compressed input datasets.
7. The tool must avoid any preprocessing/conversion of the input datasets (compressed or not) and reference genome files before being copied to HDFS.
8. The tool must be scalable, especially when processing large datasets. To do so:
  - Disk overheads should be reduced to the bare minimum (e.g., extra copies from HDFS to local disk and vice versa).
  - Any other extra overhead incurred by the Hadoop framework (e.g., data shuffling) should be avoided (if possible).

It is obvious that the underlying aligner plays a key role in fulfilling requirements 1-3. Rather than implementing a short read aligner from scratch, we have integrated HISAT2 in Hadoop in a similar way to previous tools. As mentioned before, HISAT2 has been selected because of its memory efficiency and speed features according to [23], while it is also more accurate than its popular predecessor (TopHat2). Furthermore, HISAT2 provides single- and paired-end alignment of FASTQ/FASTA datasets and produces the output in SAM format. The rest of desirable requirements heavily depend on the *HSRA* design on top of Hadoop and on how HISAT2 has been effectively integrated into it. Next sections describe *HSRA* in more detail to show how features 4-8 have been achieved.

### 4.1 HSRA overall design

*HSRA* has been designed as a command-line tool that receives as input arguments those common parameters also needed when working with any standalone aligner. For instance, the path to the input sequence and genome index files are mandatory arguments. On the one hand, the input sequence files in FASTQ/FASTA format must be stored in HDFS so that they can be processed by Hadoop in a distributed way. On the other hand, the distribution of the genome index files does not provide any performance benefit as all the aligner tasks require to load the entire genome in memory. So, the index files can be either shared among all computing nodes using a shared file system (e.g., NFS) or stored locally in the same path of all nodes. Another mandatory argument for *HSRA* is the estimated memory needed to run a single instance of the aligner taking into account the reference genome being used. The submission of the MapReduce jobs to the Hadoop cluster to perform the read alignment is facilitated by the *hsrarun* command included in the *HSRA* bundle distribution. To help non-expert users, our tool includes a user’s guide that details all the input arguments for *hsrarun*, provides compilation and execution instructions, and describes advanced configuration options.

The *HSRA* design has been focused on avoiding any modification of the original HISAT2 source code (requirement 4). For this reason, *HSRA* executes the alignment algorithm from the map tasks as an external process. To properly interact with the underlying aligner, our tool needs to use some default command-line options of HISAT2 that do not usually change between different releases. In this way, *HSRA* can be considered version-agnostic regarding HISAT2, being not limited to using a particular version (first part of requirement 5). In the unlikely event that any of the options required by *HSRA* were changed in future HISAT2 releases, our tool can be adapted through a specific configuration file included in the distribution, thus increasing version portability even more (second part of requirement 5).

### 4.2 HSRA workflow

Basically, the *HSRA* workflow consists of two main stages: (1) If the input datasets are not already stored in HDFS, they must be first distributed over the nodes of the cluster by uploading them to HDFS. In this stage, the datasets are partitioned into a variable number of data blocks according to the block size configured for HDFS (e.g., 256 MiB). (2) A MapReduce job is submitted to the Hadoop cluster using the *hsrarun* command provided by *HSRA* to perform the read alignment. In this stage, multiple instances of the HISAT2 aligner are executed in parallel over the nodes of the cluster, with each aligner task processing the input split assigned to it (by default, there is one input split per HDFS data block). Actually, the first stage is a common prerequisite for any Hadoop-based tool, so we will focus on the read alignment stage from now on.

It is important to remark that *HSRA* is specifically oriented to those users who perform their RNA-seq analysis on Big Data platforms. Consequently, they are encouraged to take advantage of downstream analytical tools that are able to perform further data processing directly on HDFS. Representative examples of such tools are the Spark-based implementation of the GATK toolkit [53] and ADAM [54], which implements a variant calling pipeline on top of Spark. Otherwise, the output files of each aligner task must be merged into a single SAM output file by performing a copy-merge operation at the HDFS level, which is an optional step in *HSRA*. Next, this file must be copied from HDFS to the local file system for further processing, incurring high disk overhead. Nevertheless, note that this is not the common use case for Big Data tools such as *HSRA*.

One of the the main advantages of the *HSRA* workflow compared to previous Hadoop-based tools is that the input sequence files can be stored and processed directly on HDFS without any previous preprocessing/conversion step (requirement 7). Moreover, these datasets can be in compressed format supporting the gzip and bzip2 codecs (requirement 6). To do so in an efficient way, we have developed the Hadoop Sequence Parser (HSP) library [55] that allows to process FASTQ/FASTA datasets (compressed or not) directly from HDFS both for single- and paired-end reads. Although the source code of this library was first strongly coupled with the *HSRA* project, it has now been released as a standalone open-source library to make it publicly available for other bioinformatics applications. In fact, we have also redesigned our MapReduce Duplicate Removal (MarDRe) tool [56] to make use of HSP for improved performance, which shows the usefulness, applicability and flexibility of our approach. The motivation to implement such a library and some details about its overall design are described next.

### 4.3 The Hadoop Sequence Parser (HSP) library

In the context of Hadoop, the access to the input files stored in HDFS is managed by an appropriate implementation of the abstract *InputFormat* class: *FileInputFormat*. This class defines how these input files are divided into logical chunks or input splits, each of them to be processed by an individual map task. The split size is configurable by the user for each MapReduce job, so it can be used to control the total number of map tasks that are executed. Note that a split is a logical division of the input data whereas an HDFS block is a physical division. In fact, the HDFS block size is used as the default split size if not specified by the user (i.e., there is one input split per HDFS block by default). An *InputFormat* class must also provide the corresponding *RecordReader* implementation to extract input records from the logical split, and is in charge of respecting record boundaries and presenting a record-oriented view of the split to the map tasks.

In the context of *HSRA*, the input sequence files must be properly parsed when processed with Hadoop taking into account the specific structure of the FASTA/FASTQ text-based formats. Unfortunately, the built-in *FileInputFormat* implementations provided by Hadoop for processing text-based files (e.g., *TextInputFormat*) are not able to handle those sequence formats straightforwardly. Hadoop is designed to process line-based text formats where identifying individual records is simple as line boundaries are denoted by newline characters (i.e., one record per line). However, FASTA/FASTQ formats are text-based files that involve multiple lines per sequence. One simple but inefficient way to solve this issue is to convert the sequence files into the appropriate line-by-line format required by Hadoop (i.e., one sequence per line) and then copy the converted files to HDFS. As mentioned in Section 3, this has been the preferred approach used by most of the previous tools based on Hadoop (e.g., BigBWA, CloudAligner, DistMap). Another approach consists in using specialized libraries that implement specific routines to parse FASTQ/FASTA files in Hadoop. These libraries provide custom implementations of the *FileInputFormat* and *RecordReader* classes, and Hadoop-BAM [46], BioPig [57] and FASTdoop [58] are available alternatives. These Java libraries allow to read single-end datasets in FASTQ/FASTA formats directly from HDFS. However, none of them provide specific support for paired-end datasets and this is the reason why Halvade, which internally uses Hadoop-BAM, still requires a preprocessing step for paired-end datasets. Furthermore, Hadoop-BAM and FASTdoop do not support compressed datasets. BioPig provides this support, but it has proved to be the most inefficient library according to [58].

To the best of our knowledge, HSP is the first library that provides specific support for both single- and paired-end datasets (compressed or not), which allows *HSRA* to avoid any conversion of the input files (requirements 6-7). Basically, HSP includes two abstract classes at the top of the hierarchy that extend the *FileInputFormat* and *RecordReader* classes from Hadoop: *SingleEndSequenceInputFormat* and *SingleEndSequenceRecordReader*, respectively. These classes are the templates to support single-end datasets, which are formed by a single input file, providing specific implementations for FASTQ (*FastQInputFormat*, *FastQRecordReader*) and FASTA (*FastAInputFormat*, *FastARecordReader*) in a similar way to previous libraries. To support paired-end datasets, HSP takes advantage of their special structure: the two ends of a paired read are distributed in two separate files with one of them containing the forward (or “left”) read and the other one containing the corresponding reverse (or “right”) read. Note that there is a one-to-one mapping between the forward and reverse reads of each sequence. If each file were separately handled as a single-end dataset, their corresponding input splits would keep the one-to-one correspondence. So, HSP supports paired-end datasets by providing an appropriate *PairedEndSequenceInputFormat* class together with a custom *InputSplit* implementation: *PairedEndCompositeInputSplit*. This class allows to create composite input splits formed by the two underlying file splits coming from each input file, keeping the aforementioned one-to-one mapping. Finally, the *PairedEndSequenceRecordReader* class uses two of the underlying record readers for single-end datasets to parse the reads from each of the input splits and merge their contents, thus providing a single record to the map task that contains both ends. It is worth mentioning that our approach does not interfere with the data-aware capabilities of Hadoop. The scheduler will still try its best to place map tasks on the nodes where both splits that make up a composite split reside (or at least one of them), minimizing network traffic. This is possible due to the location information provided by the *PairedEndCompositeInputSplit* class through the *getLocations()* method, which must be implemented by any *InputSplit* subclass.

Regarding the data types of the <key,value> pairs provided by HSP, both single- and paired-end record readers generate the following format: <LongWritable,Text>. In single-end mode, the key is a unique self-generated identifier for each read within the split and the value is the text-based content of the read (e.g., read identifier, bases and qualities for FASTQ). In paired-end mode, the key provides the length (in bytes) of a single read in the pair and the value is the merged content of both reads. If needed, users can obtain the “left” and “right” reads separately by splitting the value (i.e., the text) using the provided key. These formats for the <key,value> pairs have been chosen to make HSP agnostic of *HSRA* so that it can be used by any other Big Data framework compatible with the Hadoop input formats.

Once the HSP library has been designed to feed the map tasks with appropriate key-value pairs from the input datasets in an efficient way, the read alignment step can start. Next sections describe how this step has been implemented in *HSRA* for single- and paired-end alignment.

### 4.4 Single-end alignment

The single-end mode has been implemented using a map-only job, thus avoiding any data sorting and shuffling overhead incurred by the Hadoop framework (requirement 8(b)). By default, one map task is generally launched per input split in a Hadoop job. In order to increase the flexibility and user-friendliness of our tool, *HSRA* accepts the number of aligner instances to be executed per node through a command-line option. The total number of map tasks (i.e., aligner instances) launched in a job is internally managed by *HSRA*, which calculates the appropriate split size to create as many input splits (i.e., map tasks) as needed. The number of map tasks per node is then controlled by requesting the necessary memory resources from YARN for each task. These memory requirements are based on the available memory per node as configured in YARN and the estimated memory per aligner as indicated by the user. Note that all this configuration is automatically done by *HSRA* and is completely transparent to the user.

**Fig 2:**
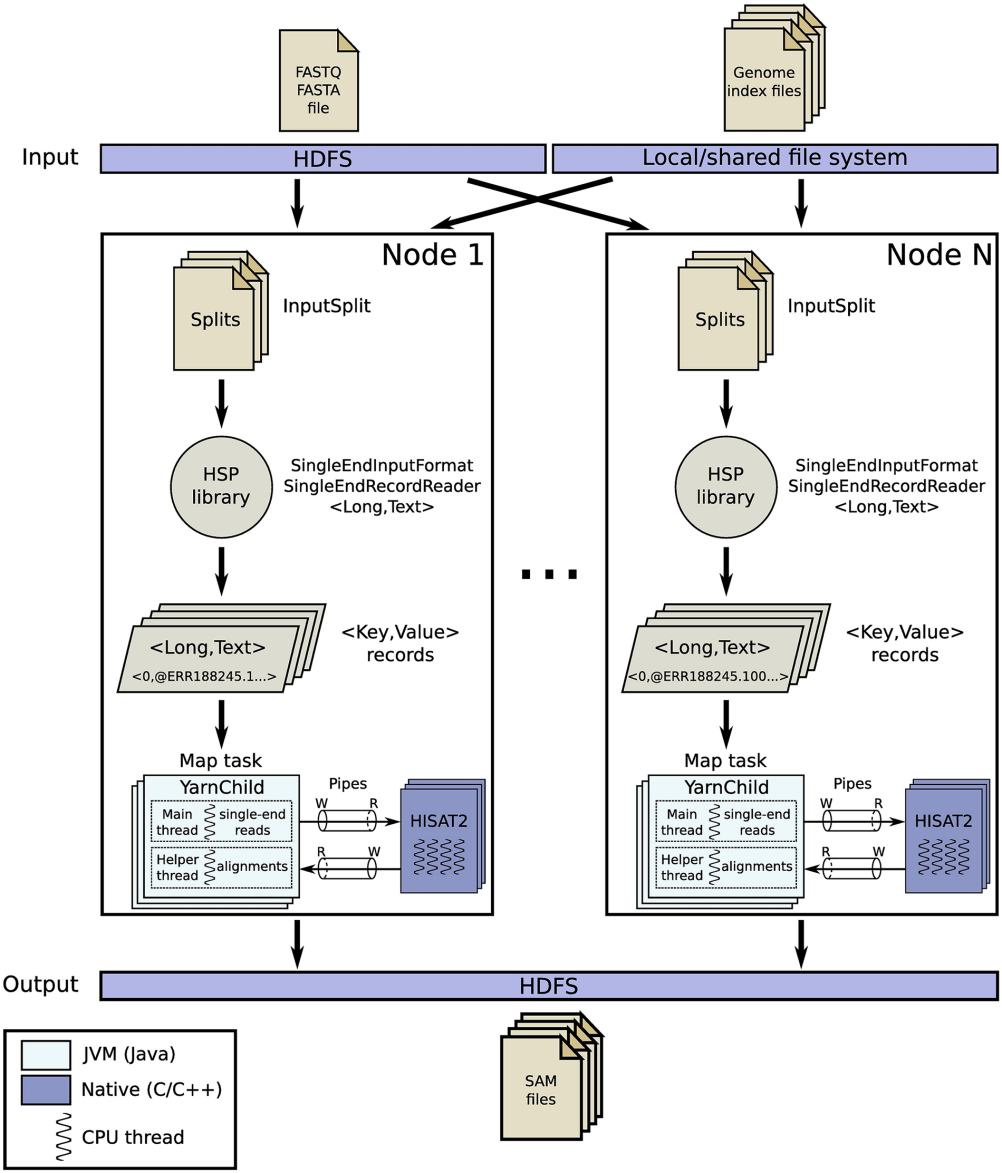
Overview of the HSRA workflow for single-end alignment. This mode executes a map-only job taking advantage of the HSP library to parse the reads directly from HDFS. Native pipes are used for efficient IPC communication between Hadoop and HISAT2.

A high-level overview of the *HSRA* workflow for single-end alignment is depicted in Fig 2. The input splits are read from HDFS using the HSP library, which parses them into key-value pairs representing single-end reads that feed the map tasks. During the *setup* method of the map phase, each map task executes a single instance of HISAT2 as an external process. This instance is in charge of aligning the reads of its corresponding input split to the whole reference genome. Next, the *map* method provided by *HSRA* is called for each input key-value pair (i.e., each read) generated by HSP. These reads are sent to the underlying aligner by using an Inter-Process Communication (IPC) mechanism to avoid any disk overhead (requirement 8(a)), as described in Section 4.4.1. Finally, the *cleanup* method of the map phase is in charge of destroying the aligner when finished. The number of threads that the underlying aligner can use to speed up the computation can also be provided as input argument to *HSRA*. One clear advantage of this design is that the alignment step can be performed using a two-level hybrid parallelization. On the one hand, several map tasks are executed across the cluster, with several tasks per node if desired. On the other hand, each map task can parallelize the alignment using several threads to exploit the multithreaded capabilities of HISAT2.

The interaction between the map task, which runs as a Java process called *YarnChild* in a Java Virtual Machine (JVM), and the underlying aligner HISAT2, which runs as a separate system process launched from the JVM, is far from straightforward. Note that, for performance reasons, state-of-the-art aligners are usually written in natively compiled languages (e.g., HISAT2 is implemented in C++), while Hadoop and HDFS are implemented in Java. So, these aligners cannot read/write directly from/to HDFS unless their source code were modified. However, they still require that the input reads to be aligned are either stored in a file or fed through the standard input stream (i.e., stdin). The problem is that the input splits are parsed from HDFS during the map phase, so the reads reside in the JVM memory space. As mentioned in Section 3, most of the previous tools (e.g., BigBWA, SparkBWA, DistMap, Halvade) have overcome this issue through the local file system by copying the reads parsed from HDFS to a file stored in local disk, and then passing the path to this file as an input argument to the aligner, thus incurring disk overhead. In a similar way, aligners generally write the alignments either to an output file or to the standard output stream (i.e., stdout). The common approach is again to copy the output file from local disk to HDFS in the map task when the aligner has finished. As mentioned before, *HSRA* avoids any disk overhead during the alignment step by using an IPC mechanism that is based on named pipes, as described next.

#### 4.4.1 Efficient IPC mechanism between Hadoop and HISAT2

A named pipe (or FIFO) provides an efficient one-way IPC channel between two separate processes running on the same node, without incurring the performance penalty of involving the disk or the network stack. In fact, the communication using a named pipe is performed through a memory buffer that exists inside the kernel space, with one process acting as reader and the other one as writer. The underlying implementation ensures that a named pipe stays in memory rather than being written to disk. This concept is an extension of the traditional pipe that lasts beyond the life of the process. The “name” of a named pipe is actually a file name within the file system that appears as it were a regular file. Using this mechanism, two separate processes can be attached to a named pipe by its name (i.e., its path) for doing efficient IPC through the file system, without incurring any disk overhead.

Unfortunately, the JVM does not currently provide any routine to create named pipes from Java code. Instead, *HSRA* must resort to native APIs (e.g., *mkfifo*). Such APIs can be accessed from Java via the Java Native Interface (JNI), which allows the execution of native code (e.g., C/C++) inside the JVM. In *HSRA*, the map task first creates two named pipes through JNI for the input and output files required by HISAT2 in single-end mode. Once created, standard Java routines for file I/O (e.g., open, read, write, close) can be performed over named pipes in the same way as with regular files. Next, the map task launches the aligner with the paths of the input and output pipes as arguments. So, HISAT2 acts as reader while the map task must open the input pipe as writer. The reads to be aligned are streamed (i.e., written) by the map task to the input pipe as they are read from the split stored in HDFS, while the aligner is concurrently reading from the pipe (see Fig 2). In a similar way, the map task opens the output named pipe as reader to consume the output alignments produced by HISAT2. As any other standard MapReduce application, these alignments are written to HDFS by *HSRA* during the map phase as output <key,value> pairs using the following format: <NullWritable,Text>. So, our pipe-based mechanism avoids the use of any intermediate file on local disks as, unlike previous tools, the output from the aligner is directly written to HDFS. Finally, note that the map task launches a helper thread to consume the data coming from the aligner at the same time that the main thread is feeding the aligner through the input pipe. As mentioned before, named pipes are internally implemented using a memory buffer, whose maximum size is limited. When this buffer is full, any write operation to the pipe blocks until data are consumed. So, the helper thread is needed to consume the data as soon as they are produced by the aligner, avoiding any possible deadlock that would occur if both pipes were managed by a single thread.

### 4.5 Paired-end alignment

The paired-end mode follows the same overall design as single-end, thus taking advantage of the two-level parallelization and the efficient IPC communication between Hadoop and HISAT2 through named pipes. In fact, the number of aligners to execute and the number of threads per aligner can also be specified for this mode. Currently, *HSRA* provides two different approaches for processing paired-end datasets, both implemented using a single MapReduce job. The first one, which can be considered a naive approach, uses the single-end support from the HSP library and performs a reduce-side join in order to pair the reads from the two input files required in this mode. The second approach takes advantage of the specific support provided by HSP for paired-end datasets to perform the join on the map side, thus executing a map-only job. The goal of providing two different implementations is to show the performance benefits obtained due to using this specific support only available in the HSP library. Each approach is detailed next.

#### 4.5.1 Reduce-side join approach

**Fig 3:**
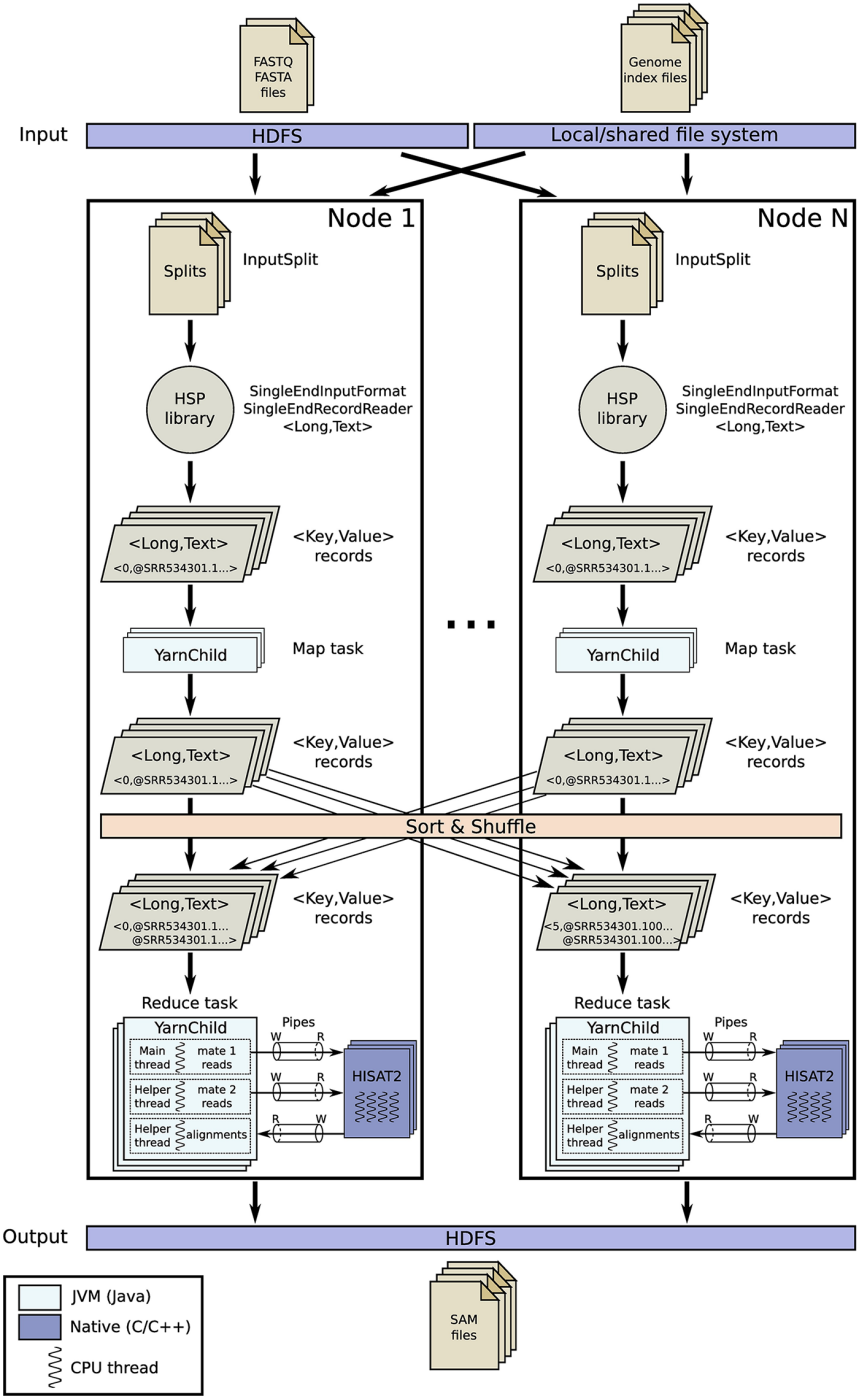
Overview of the HSRA workflow for paired-end alignment using the reduce-side join approach. This approach executes a MapReduce job using the single-end support provided by the HSP library, where a reduce-side join is needed to obtain the paired-end reads. Native pipes are used for efficient IPC communication between Hadoop and HISAT2.

The overall workflow of this approach is shown in Fig 3. Using the single-end support from HSP, each map task processes a split from one of the two input files required in the paired-end mode.

Before the paired-end alignment can actually be performed, the reads from the *i*-th split of the first input file must be joined (i.e., paired) with the corresponding ones from the *i*-th split of the second input file. To do so, map tasks parse their corresponding splits from HDFS as in single-end mode, but they emit key-value pairs where the key is the unique identifier within the split generated by HSP and the value is the read parsed as text. As explained in Section 4.3, there is a one-to-one correspondence between the forward and reverse reads in paired-end datasets. So, the identifier of the *j*-th read from the *i*-th split of the first input file will be the same as that of the *j*-th read from the *i*-th split of the second one. The grouping-by-key operation performed by the MapReduce data engine between the map and reduce phases acts as a reduce-side join, where both the forward and reverse reads of each paired-end sequence are sent to the same reduce task. Remind that the input of a reduce task is a single list that contains all the values (2 in this case) with the same key. In this way, the paired-end alignment can be performed during the reduce phase. First, reduce tasks create two named pipes for both input files required by HISAT2, and a third one for the output file. Next, each reduce task launches a single instance of HISAT2. The paired-end reads are obtained from the input key-value pairs received by a reduce task, which are streamed to the input pipes. The main thread is in charge of feeding the first input pipe being read by HISAT2, while a helper thread does the same for the second one. Another helper thread is also needed to receive the output data from the aligner and write them to HDFS to avoid any deadlock, as in single-end mode.

This approach does not require any specific support for paired-end datasets from the HSP library. Instead, it relies on the single-end support and leverages the underlying MapReduce engine to perform the join-like operation required to merge both ends of a paired-end sequence in the reduce side, which executes the alignment step. However, sorting and data shuffling mechanisms between the map and reduce phases involve disk and network overheads, which can limit performance and scalability.

#### 4.5.2 Map-side join approach

**Fig 4:**
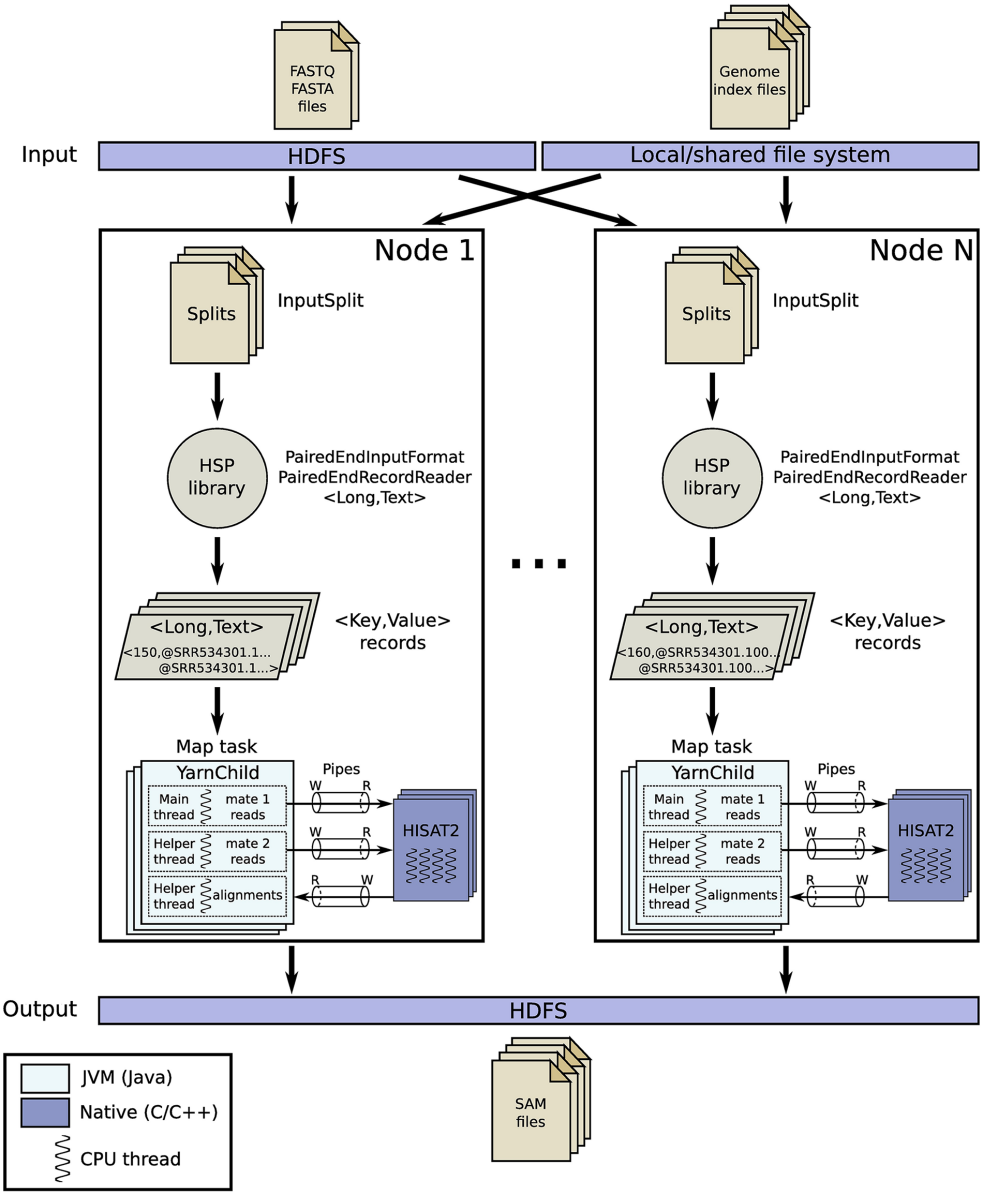
Overview of the HSRA workflow for paired-end alignment using the map-side join approach. This approach allows avoiding any data shuffling by executing a map-only job thanks to the specific support for paired-end datasets provided by the HSP library. Native pipes are used for efficient IPC communication between Hadoop and HISAT2.

This approach takes full advantage of the HSP library to process paired-end datasets in a more efficient way. The overall workflow is depicted in Fig 4. As can be seen, the custom input format and record reader provided by HSP for paired-end reads allows *HSRA* to perform the alignment step during the map phase, thus executing a map-only job that avoids data sorting and shuffling overheads (requirement 8(b)).

As explained in Section 4.3, HSP can transparently feed the map tasks with appropriate key-value pairs for paired-end alignment, where the key provides the length of each end of a paired read and the value contains the merged content of both ends. The join-like operation that merges both ends of a paired read from the two input files occurs in the map side when reading the composite splits from HDFS, which have been previously created by the HSP library. During the map phase, the map tasks use the key to split the value (i.e., a paired-end sequence) into separate forward and reverse reads. These separate reads are then streamed to HISAT2 through two named pipes in the same way as in the previous approach, with the output from the aligner being also handled through an additional named pipe.

## 5 Results and discussion

In this section, *HSRA* is evaluated on a 16-node multi-core cluster using Hadoop version 2.7.3 and HISAT2 version 2.1.0. Each computing node consists of two Intel Xeon E5-2660 octa-core processors at 2.2 GHz (i.e., 16 cores per node), 64 GiB of memory and one 800 GiB local disk intended for both HDFS and intermediate data storage during the execution of Hadoop jobs. The nodes are interconnected through an InfiniBand FDR network (56 Gbps). The system runs Linux CentOS 6.8 with kernel 2.6.32-642, and the JVM version is Oracle JDK 1.8.0_144. Regarding HDFS settings, the block size and the replication factor were set to 256 MiB and 3, respectively. Four publicly available datasets (stored in HDFS) have been used to evaluate *HSRA*, named after their accession numbers in the NCBI sequence read archive (see Table 1 for their main characteristics). We have selected datasets with different representative sizes (from 23 to 96 GiB) and read lengths (76 and 101 base pairs). The alignments were performed for single- and paired-end reads to the reference human genome *hg38*, whose index files are available in the local disk of each node. Finally, the results shown in this section correspond to the median runtime for a set of 10 executions for each dataset.

**Table 1:** Input datasets used in the experimental evaluation.

Tag	Name	Instrument model	Organism	#Reads	Read length	Size
SRR1	SRR534301	Illumina HiSeq 2000	Homo sapiens	108.75 M	101 bp	24 GiB
DRR1	DRR021368	Illumina HiSeq 2500	Homo sapiens	289.15 M	101 bp	96 GiB
SRR2	SRR317060	Illumina Genome Analyzer II	Homo sapiens	110.47 M	76 bp	23 GiB
SRR3	SRR567455	Illumina HiSeq 2000	Homo sapiens	251.88 M	76 bp	45 GiB

Characteristics of the public datasets used in the evaluation of *HSRA*, named after their accession numbers in the NCBI sequence read archive.

**Fig 5:**
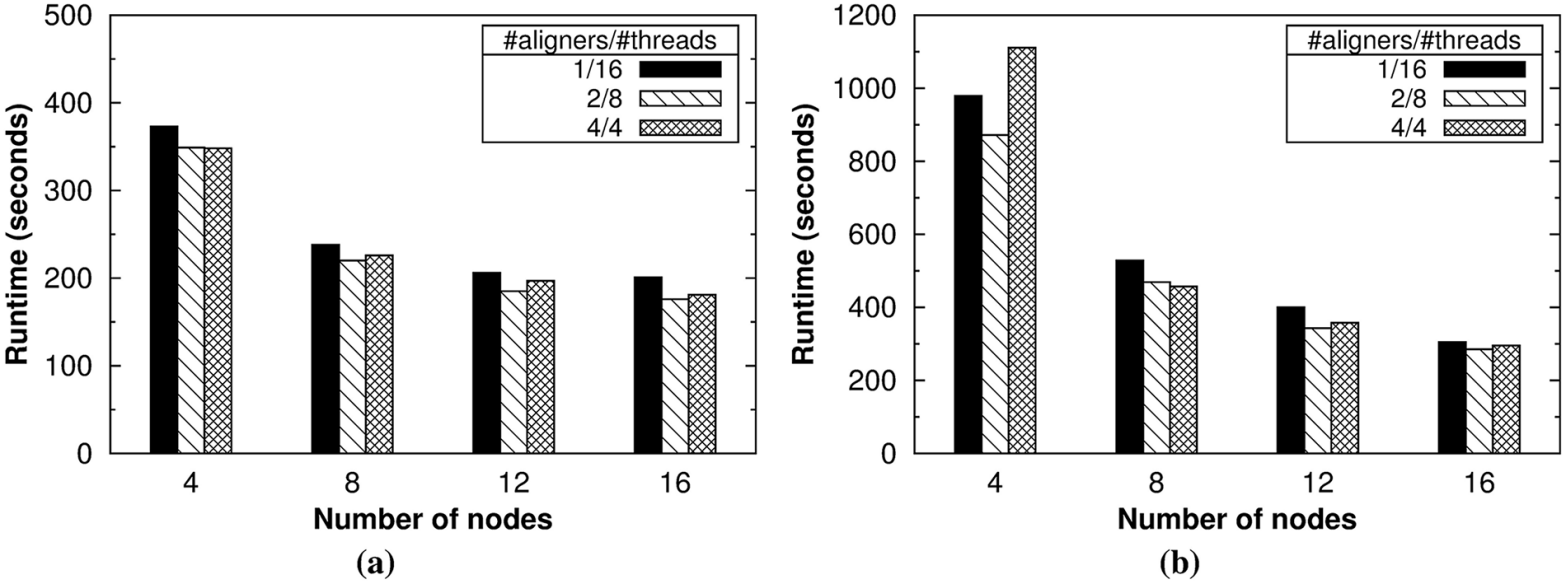
Experimental results for single-end alignment. Runtime results obtained by *HSRA* when varying the number of nodes using the (a) SRR1 and (b) DRR1 datasets.

**Fig 6:**
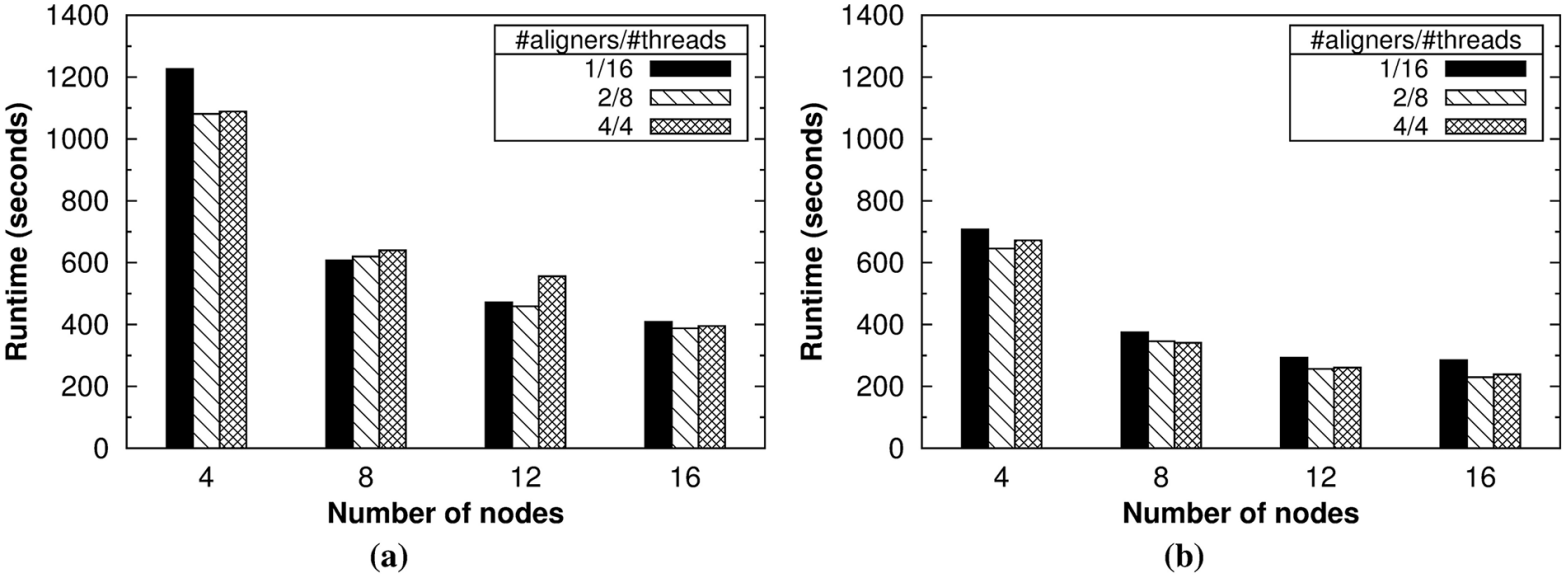
Experimental results for paired-end alignment (SRR1 dataset). Runtime results obtained by *HSRA* when varying the number of nodes using the (a) reduce-side and (b) map-side join approaches.

**Fig 7:**
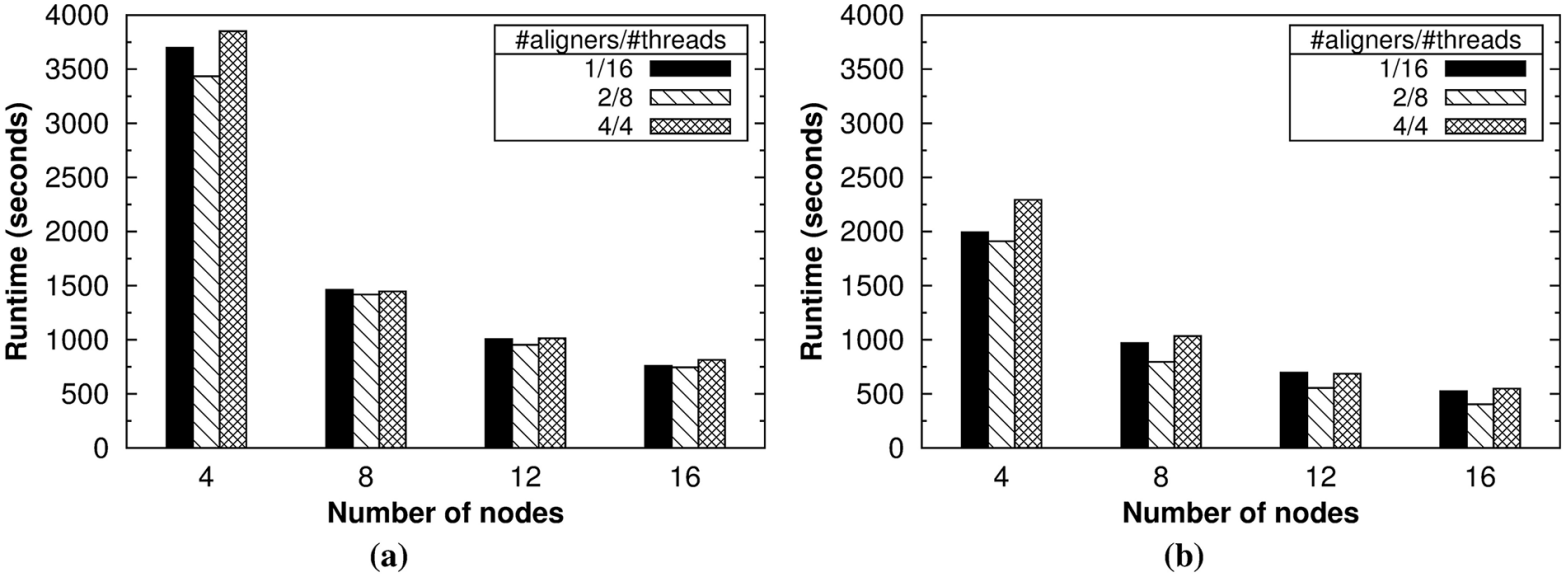
Experimental results for paired-end alignment (DRR1 dataset). Runtime results obtained by *HSRA* when varying the number of nodes using the (a) reduce-side and (b) map-side join approaches.

As explained in Section 4.4, the flexibility of our tool allows the user to easily set via command-line options the number of aligner instances to execute per node (*-na*) and the number of threads to use per aligner (*-nt*). *HSRA* also provides two different approaches for paired-end alignment described in Section 4.5. So, the experimental evaluation started by finding the best configuration for the number of aligners and threads, as well as the best approach for paired-end mode. For these experiments, the datasets with the largest read lengths were used: SRR1 and DDR1 (see Table 1). Fig 5 shows the runtime for the single-end alignment of both datasets for different configurations using 4, 8, 12 and 16 nodes, while Figs 6 and 7 provide the same results for the paired-end alignment of the SRR1 and DRR1 datasets, respectively, using both approaches.

The first conclusion that can be drawn from these results is that the intermediate configuration for the number of aligners and threads is the best performer overall: two aligner instances per node and eight threads per aligner on this system. Nevertheless, the performance differences between configurations are generally small, especially when using the largest number of nodes. As a rule of thumb, non-expert users can select as many aligner instances as processors per node and as many threads as cores per processor. The second conclusion is that the map-side join approach for paired-end alignment significantly outperforms the reduce-side counterpart for all configurations and number of nodes (see Figs 6 and 7). In fact, the map-side join is on average 1.8 times faster than its counterpart for the aforementioned intermediate configuration, which clearly shows the performance benefits provided by the HSP library when using its specific support for paired-end datasets. Finally, the third conclusion is that our tool provides good scalability overall, especially for the largest dataset (i.e., DRR1). From now on, all the experimental results shown in this section have been obtained with the best configuration for *HSRA*.

### 5.1 Performance comparison with DistMap

Big Data users that perform their RNA-seq data analyses on HDFS can take advantage of the performance benefits provided by *HSRA* when mapping very large datasets. In order to accurately measure such benefits and provide a fair comparison with previous Big Data mapping tools, they should ideally use the same underlying aligner. This would ensure that the raw performance and memory consumption of the alignment step remains the same for all the tools. So, any performance difference between them could be directly attributed to their implementation on top of the underlying aligner, and not due to using different alignment algorithms. However, *HSRA* is, up to our knowledge, the first publicly available distributed tool based on HISAT2. As mentioned in Section 3.2, DistMap [50] is one of the Hadoop-based tools that supports several aligners for RNA-seq data, TopHat2 being one of them, which is the predecessor of HISAT2. It was therefore feasible to adapt DistMap by modifying its source code to use HISAT2 instead of TopHat2, and thus provide a fair comparison with our tool. Note that the DistMap results shown in this section do not include the extra time required for the preprocessing step to prepare the input datasets. We report the time needed to perform only the alignment stage, which provides a best-case scenario for DistMap performance.

Table 2 shows the experimental results for DistMap and *HSRA* for the single- and the paired-end alignment of the four datasets using 4, 8, 12 and 16 nodes. These results validate our design as they prove that our tool is on average around 2.3 times faster than DistMap when using the same underlying aligner. On the 16-node cluster, the maximum speedups of *HSRA* over DistMap are 3.70x and 3.30x for single-end (SRR3) and paired-end (SRR2) modes, respectively. The main reason for this performance improvement is that *HSRA* does not incur any disk overhead during the alignment step (requirement 8(a) in Section 4) by streaming the input reads from HDFS to HISAT2 through named pipes, while consuming its output data in the same way. Instead, DistMap first copies the input reads from HDFS to local files stored in disk and passes their paths to HISAT2 to perform the alignment, while the SAM output files are also first stored in local disk and then copied to HDFS. It is important to remark that the results for DistMap do not include the preprocessing of the input datasets, which would further increase its runtimes, while *HSRA* avoids any preprocessing step by using the HSP library.

**Table 2:** Experimental results for DistMap and HSRA using HISAT2.

Alignment	Nodes	SRR1		DRR1		SRR2		SRR3		Speedup
		DistMap	*HSRA*	DistMap	*HSRA*	DistMap	*HSRA*	DistMap	*HSRA*	
Single-end	4	734	349	1564	872	989	440	4301	1977	2.09x
	8	467	220	880	469	591	242	1923	880	2.13x
	12	419	185	692	343	511	189	1715	645	2.45x
	16	358	176	495	285	428	135	1621	438	2.81x
Paired-end	4	1479	646	3357	1910	2021	1072	12162	6592	1.86x
	8	785	346	1681	796	1019	575	5350	2415	2.14x
	12	496	257	1285	555	870	326	3123	1455	2.23x
	16	401	230	784	423	751	228	2760	1069	2.41x

Runtime results (in seconds) for DistMap and *HSRA* for single- and paired-end read alignment. Both Hadoop-based tools use HISAT2 as the underlying aligner. The speedups shown are the average ratio of DistMap runtimes to *HSRA* ones for all the datasets and each cluster size.

The results shown in this section reinforce the main motivation of this paper: the design of existing Big Data tools (e.g., DistMap) cannot take full advantage of state-of-the-art aligners such as HISAT2, and a new tool implemented from scratch to overcome this issue is indeed advisable.

### 5.2 Performance comparison with HISAT2

Table 3 shows the runtimes of DistMap and *HSRA* using 16 nodes (i.e., 256 cores) and compares them with those of HISAT2 on a single node using all the available cores (16). This scenario allows measuring the maximum performance benefits of using Hadoop-based tools when aligning a single dataset on HDFS by distributing the workload across the cluster. As can be seen, DistMap and *HSRA* provide significant speedups over HISAT2: up to 6.45x and 15.54x for single-end alignment, respectively, reducing runtimes to a few minutes. As expected, aligning paired-end datasets is significantly more computationally intensive than single-end ones. This fact allows DistMap and *HSRA* to further improve their speedups up to 10.13x and 18.77x, respectively.

**Table 3:** Experimental results for DistMap and HSRA (16 nodes) vs HISAT2 (1 node).

Alignment	Dataset	HISAT2	DistMap		HSRA	
		Runtime	Runtime	Speedup	Runtime	Speedup
Single-end	SRR1	1171	358	3.27x	176	6.65x
	DRR1	3193	495	6.45x	285	11.20x
	SRR2	1508	428	3.52x	135	11.17x
	SRR3	6806	1621	4.20x	438	15.54x
Paired-end	SRR1	3281	401	8.18x	230	14.27x
	DRR1	7939	784	10.13x	423	18.77x
	SRR2	4009	751	5.34x	228	17.58x
	SRR3	19832	2760	7.19x	1069	18.55x

Runtime results (in seconds) for HISAT2 on one node and results for DistMap and *HSRA* on a 16-node Hadoop cluster when aligning each dataset. The speedups shown are the ratio of HISAT2 runtimes to DistMap and *HSRA* ones.

Although the scalability of HISAT2 is limited to one node when aligning a single dataset, users can also execute one HISAT2 instance per node to simultaneously align multiple datasets on a cluster. Table 4 shows the runtime of HISAT2 when aligning the four datasets on four nodes (i.e., one dataset per node). In this scenario, the HISAT2 performance corresponds to the maximum runtime of the most compute-intensive alignment on this system (i.e., SRR3). These results are compared with the runtimes of DistMap and *HSRA* when aligning the four datasets on a 4-node Hadoop cluster, so using the same amount of computing resources. These tools must execute four Hadoops jobs in total (i.e., one job per dataset). Basically, their performance corresponds to the sum of the alignment times for each dataset on a 4-node Hadoop cluster (see Table 2) plus the overhead of launching each Hadoop job (around 20 seconds per job for our testbed). As expected, the performance benefits of Hadoop-based tools are reduced. In fact, HISAT2 outperforms DistMap for single-end alignment, while the speedup of DistMap over HISAT2 for paired-end mode is negligible (1.04x). Nevertheless, *HSRA* is still around 2 times faster than HISAT2 in this scenario. These results prove that our tool is the suitable replacement of DistMap when performing RNA-seq analyses on HDFS, while HISAT2 would be the preferred choice otherwise.

**Table 4:** Experimental results for HISAT2, DistMap and HSRA (4 nodes).

Alignment	HISAT2	DistMap		HSRA	
	Runtime	Runtime	Speedup	Runtime	Speedup
Single-end	6806	7673	0.89x	3728	1.83x
Paired-end	19832	19098	1.04x	10302	1.93x

Runtime results (in seconds) for HISAT2, DistMap and *HSRA* when aligning all the datasets using four nodes. The speedups shown are the ratio of HISAT2 runtimes to DistMap and *HSRA* ones.

### 5.3 HSRA correctness

We have tested the correctness of *HSRA* using the SRR1 dataset in single- and paired-end mode. To assess the impact of distributing the input reads across the cluster, we have performed these experiments using the minimum and maximum number of nodes considered in this work (4 and 16, respectively). The procedure was the following: (1) the output SAM files generated by *HSRA* were first merged into a single SAM file, one for each alignment mode, to be later processed using the SAMtools package [52], as this software does not support HDFS; (2) the two merged SAM files were then copied from HDFS to local disk and analyzed with SAMtools to extract meaningful mapping statistics from the alignments contained in them; (3) these results were compared with those obtained by HISAT2 when executed as a standalone tool; and (4) the uniquely aligned reads, which represent more than 90% of the mapped reads for the SRR1 dataset, were further analyzed to obtain the number of misaligned reads generated by *HSRA* (i.e., reads mapped to a different chromosome and/or position).

Table 5 shows the most relevant metrics obtained by analyzing with SAMtools the corresponding SAM files of HISAT2 and *HSRA*. Overall, we found very small differences for most of the metrics. These differences are due to the default hybrid approach implemented by HISAT2, explained in Section 2.1.1. This approach collects splice sites as it processes the input reads. These sites are then used when aligning later reads in the same execution. So, when distributing the input dataset across the cluster, each aligner task only processes part of the data. Therefore, more/less/different splice sites (there is no way to know that) may be collected by each aligner task. This fact mainly affects the ability of HISAT2 to search for multiple distinct alignments for each read, as indicated by the reported number of valid alignments and secondary (non-primary) alignments (i.e., *HSRA* reports less multimapped reads than HISAT2). According to the number of unmapped reads, *HSRA* introduces a small percentage (<1%) of false-positive alignments, which are uniquely mapped by our tool. This in turn increases the total number of reads mapped by *HSRA* and thus its overall alignment rates are slightly higher (<0.1%) than those obtained by HISAT2. Regarding misaligned reads, we have checked that only 0.45% and 0.75% of the uniquely aligned reads generated by *HSRA* using 4 and 16 nodes, respectively, are mapped to a different chromosome and/or position when compared to those of HISAT2.

**Table 5:** Mapping statistics for HISAT2 and HSRA (SRR1 dataset).

		HISAT2	*HSRA* (4 nodes)	Diff.	*HSRA* (16 nodes)	Diff.
Single-end	Alignments	124.16 M	123.60 M	-0.45%	123.24 M	-0.74%
	Secondary alignments	22.67 M	22.10 M	-2.51%	21.71 M	-4.23%
	Reads unmapped	7.25 M	7.22 M	-0.41%	7.21 M	-0.55%
	Reads mapped	101.49 M	101.52 M	0.03%	101.53 M	0.04%
	Uniquely mapped reads	92.30 M	92.77 M	0.51%	93.10 M	0.87%
	Alignment rate	93.33%	93.36%	0.03%	93.37%	0.04%
	Bases mapped	10,209.15 M	10,212.24 M	0.03%	10,212.30 M	0.03%
	Mismatches	48.10 M	48.25 M	0.31%	48.31 M	0.44%
	Error rate	0.004711	0.004725	0.30%	0.004731	0.42%
	Average quality	34.10	34.10	0.00%	34.10	0.00%
	Average coverage	3.348	3.349	0.03%	3.349	0.03%
Paired-end	Alignments	235.46 M	233.06 M	-1.02%	231.48 M	-1.69%
	Secondary alignments	37.24 M	34.72 M	-6.77%	33.08 M	-11.17%
	Reads unmapped	19.28 M	19.17 M	-0.57%	19.10 M	-0.93%
	Reads mapped	198.22 M	198.33 M	0.06%	198.40 M	0.09%
	Reads mapped and paired	188.51 M	188.70 M	0.10%	188.82 M	0.16%
	Uniquely mapped reads	182.43 M	184.45 M	1.11%	186.19 M	2.06%
	Alignment rate	91.14%	91.18%	0.04%	91.21%	0.08%
	Bases mapped	19,937.70 M	19,949.70 M	0.06%	19,955.69 M	0.09%
	Mismatches	117.06 M	117.64 M	0.50%	118.01 M	0.81%
	Error rate	0.005871	0.005897	0.44%	0.005913	0.72%
	Average quality	32.90	32.90	0.00%	32.90	0.00%
	Average coverage	6.5384	6.5424	0.06%	6.5443	0.09%

Mapping statistics (and their differences) for HISAT2 and *HSRA* for single- and paired-end read alignment of the SRR1 dataset. These results were obtained by analyzing the output SAM files generated by both tools using the SAMtools package. For *HSRA*, two sets of results are provided for the minimum (4) and maximum (16) number of nodes considered in this work.

Finally, it is worth noting that increasing the number of nodes by a factor of 4 (from 4 to 16), and thus the number of aligner tasks executed by *HSRA*, does not widen these differences in the same proportion. Remark also that previous Big Data mapping tools [37, 39–41] that do not rely on HISAT2 have also reported small differences when verifying their correctness compared with their corresponding standalone aligners. This fact evidences that any distributed mapping tool that splits the input data into several chunks can minimally affect the output alignments one way or another depending on the underlying aligner used.

## 6 Conclusions

Recent advances in NGS technologies have established the need for fast tools to align RNA-seq reads to a reference genome. In this paper we have presented *HSRA*, a Hadoop-based tool that obtains good scalability on multi-node systems while providing comparable accuracy to its underlying aligner, HISAT2. After a preliminary review of the literature we established eight requirements that *HSRA* successfully fulfills and make our tool more flexible and interesting than the existing counterparts. For instance, we developed the HSP library as basis of *HSRA* to efficiently parse FASTA/FASTQ files from HDFS avoiding expensive preprocessing or conversion steps.

The performance of our tool has been evaluated on a 16-node Hadoop cluster using four large datasets. Our results have shown experimental evidence of significant performance improvements in terms of execution times and scalability compared to a previous Hadoop-based tool (DistMap) using the same underlying aligner. In fact, *HSRA* is up to 3.70 times faster than DistMap for the single-end alignment of a huge dataset with 251 million 76 bp reads to the human genome when using all the cluster nodes. *HSRA* is distributed as free software under the GNU GPLv3 license and is publicly available to download from http://hsra.dec.udc.es.

As future work, we aim to evaluate the performance of *HSRA* on public cloud platforms such as Amazon EMR. Furthermore, we intend to adapt our tool to exploit other Big Data frameworks such as Apache Spark.
